# Mapping International Geopolitical Agenda. Continuing National Conceptions of the Emerging European Crisis

**DOI:** 10.3389/fdata.2021.718809

**Published:** 2021-12-06

**Authors:** Claude Grasland, Etienne Toureille, Romain Leconte, Marta Severo

**Affiliations:** ^1^ FR 2007 CIST, Université de Paris, CNRS, Université Paris 1 Panthéon-Sorbonne, Paris, France; ^2^ UMR 8504 Géographie-cités, CNRS, Université Paris 1 Panthéon-Sorbonne, Université de Paris, Paris, France; ^3^ UMR 6266 IDEES, Université de Rouen-Normandie, Rouen, France; ^4^ EA 7339 DICEN-IDF, Université Paris-Nanterre, CNAM, Université Gustave Eiffel, Paris, France

**Keywords:** press international news, RSS (really simple syndication), Europe, migrant, crisis, borders and bordering, geopolitics, pandemics

## Abstract

This study proposes a geopolitical analysis of opinion dynamics based on a statistical exploration of a press dataset covering 2014–2019. This exploration questions three case studies of geopolitical and international interest: international migration, political borders, and pandemics. Through the framework of *geopolitical agenda*, the aim of this study is to question the “crisis” status of changes in the media coverage of the three topics in a cross-analysis and multilingual analysis of 20 western European newspapers. It concludes that there is a prevalence of national agendas.

## 1 Introduction

Multiple geopolitical crises have unfolded in Europe and European countries in the last decade: a migration crisis, the Ukrainian crisis, Brexit, and a crisis of representative democracies, among others. The European Union is struggling to cope with these crises. The term “crisis” abounds in public use by journalists, scientists, and politicians to the point “crisis” can appear to be the new normal.

What kind of geopolitical crisis can be observed over the period 2014–2019? Are these crises reported at the same time and with the same intensity in the media of different countries? Are they associated with the same types of events located in the same countries? Finally, to what extent can we assume the existence of a common agenda of crisis reported by media outlets from Western European countries?

In this study, we propose building a framework to analyze the production of geopolitical crises by the media from a critical perspective and to examine how a geopolitical topic becomes a crisis from the perspectives of European newspapers. A crisis appears as a heuristic concept when it is considered a social construct from the public sphere. First, the crisis is an interdisciplinary object of research, which allows several fields of research to be brought together around complex and multidimensional social objects and to go beyond the frontiers of geopolitics. Second, a crisis is a classical topic in geopolitics but can also be reformulated within the renewed theoretical framework of critical geopolitics. Since the 1990s, critical geopolitics has paid particular attention to the analysis of discourse and representation. Then, the process of analyzing geopolitical phenomena as crisis in the public sphere constitutes the subject of the critical geopolitics of crises. Third, a crisis is an operational concept in data analysis. Indeed, analyzing the public sphere using big data requires transferring the concept into methods of analysis. However, the crisis, as a rupture, lends itself well to statistical modeling.

In this study, we undertake a statistical analysis to contextualize and explore geopolitical crises in the daily press prior to in-depth thematic studies. Based on the exploration of a massive corpus of short texts (titles and first sentences of an article) could lead to more qualitative analysis of the full text of the articles or use of methods with more substantial and costly computational procedures, such as precision grammar. This study makes a novel contribution to the literature by undertaking a multilingual and comparative analysis of the agendas of three geopolitical crises over a relatively long period (2014–2019) and a large sample of media outlets located in five countries (France, Germany, Spain, Italy, and the United Kingdom). For this purpose, we use an interactive dashboard (TELEMAC) that has been developed within a European research in computational social sciences applied to opinion dynamics (Acknowledgments) to visualize and compare the spatial and temporal distribution of crises from a multidimensional perspective.

The rest of the paper is organized in four sections as follows.

1) **Conceptual framework.** First, we present the concept of *geopolitical agenda*, which could be a framework for investigating the notion of “crisis” as a way to question the changing perception of topics in the daily press. We present this conceptual framework and the related methods as a list of dimensions that should be combined in the phase of data collection and exploration. We also present three case studies of crisis (borders conflicts, international migration, and pandemics).
2) **Data collection and exploration.** Then, we present the corpus of news that has been collected, the original data source (an RSS feeds dataset from media outlet), and how these data have been enriched (e.g., addition of information related to detection of topics of geopolitical interest, and to the detection of foreign countries mentioned in the news). We also briefly present the TELEMAC interface and methodology used for the statistical analysis presented in the results.
3) **Results.** In this section, we present a selection of results obtained through the application of statistical models to each of the three topics for the entire period of observation.
4) **Discussion.** The general results of the analysis are that the salience of each topic is comparable in each country of observation, and the geopolitical agenda is mainly structured at the national level. However, we observe notable exceptions that could be identified as crisis. We provide a solution to build a comparative and multilingual analysis of the coverage of topics by several media outlets to detect the possible emergence of crisis through space and time. Second, we discuss the contribution of the analysis to the literature with respect to each case study. The study concludes with a discussion on the formulation of several hypotheses to explain the differences between crises.

## 2 Hypotheses

### 2.1 Media and Critical Geopolitics

Political geography and geopolitics have many possible definitions since their emergence as disciplines in the 19th century. Despite important theoretical transformations and the increase in the number of themes since the 1960s (from electoral geography to new critical geopolitics, including Marxist and feminist approaches), political geography has not departed much from its central themes (state, territory, and power) by raising the following issue: how the “political” is spatially constituted (Agnew et al., 2015). Like other disciplines interested in such subjects (international relations, political sociology, media studies, etc.,), political geographers have long considered the role of media in political action. The term “geopolitics” itself describes a form of political discourse that is based on space and geography and is produced by politicians to shape strategy and/or present it to the public (through propaganda and political communication). It is “a term coined by Swedish political scientist Rudolf Kjellén (1916) to capture what he saw as the geographical basis of world politics, emerged as a metonym for political geography and an expression of an organicist conception of the state and interimperialist rivalry” (Kjellén, 1916; Parker, 1985). Nowadays, this use of the media still exists. For instance, the media can be used by the state and the government to influence public opinion on a subject, as in the case of geopolitical conflicts. As noted by Boulanger, press (combined with social media) can be used as a tool by protagonists of conflict to organize, mobilize, disseminate information, notably by using the so-called “net-troll strategies,” which produce huge volumes of content to frame the agenda and the attitudes on a specific discussion (Boulanger, 2014).

In the 1990s political geography was enriched by the influence of post-modern and post-colonial academics that oriented the field toward a less conflict-oriented approach with the analysis of discourse and representation of spaces and territories (Tuathail and Agnew, 1992). Geopolitical perceptions, knowledge, and discourse about the geographical features of international relations have become a research subject (Mamadouh, 1998) based on the principle that “the functioning of geographical knowledge not as an innocent body of knowledge and learning, but as an ensemble of technologies of power concerned with the governmental production and management of territorial space.” (Tuathail, 1996). This textual shift has moved geopolitics closer to media studies. In this context, O’Tuathail (Tuathail and Agnew, 1992) proposed four theses for a narrative-based geopolitics. The first thesis considers that designating places and locations is not simply spatializing a phenomenon into a Euclidean referential but brings out a series of narratives, world visions, and appropriate political actions. As a correlate, the analysis of discourse on space is a way to obtain territorial narratives. Second, thesis proposes to define two kinds of geopolitical production: the practical type, which covers the daily action of politicians and administrators of state; and the formal type, which covers the discourse of strategic thinkers and intellectuals. This partition into two types of geopolitics was later enlarged by Dalby and Tuathail (1998), who identified a third type of geopolitics: the popular geopolitics present in mass media, cinema, novels, or cartoons, all of which contribute to the spatialization of boundaries and dangers (the geopolitical map of the world) and the geopolitical representations of self and others (the geopolitical imagination). The third thesis proposes to consider the world system as a whole, which implies a global analysis of discourse considering its embeddedness into local, national, and transnational interpretative communities. Finally, rather than state dominance, the normative power of the international stage is studied, that is, the power to bring narrative to the agenda and define the frame of interpretation. Critical geopolitics has opened up a specific field of discourse analysis focused on territorial narratives in the press to address the political framing of how to consider the world or a specific territory. We propose analyzing the geopolitical agenda of the media. Our work is in line with agenda setting theory (McCombs and Shaw, 1972). *Geopolitical agenda is defined as the spatial dimension of the agenda of political topics, which are the subject of concerns, deliberation, and action related to politics (i.e., topics addressed by the government policies).* The geopolitical agenda is considered a sub-component of the media agenda, but the limits between what is geopolitical and what is not can be very fuzzy. At first glance, a song contest or a basketball match could be considered external to the geopolitical dimension. However, research has demonstrated that the vote at the Eurovision contest could reveal fascinating proximities or antagonism between countries (Fenn et al., 2006; Gleyze, 2011). Furthermore, protests against former US president Donald Trump’s border policy utilized NBA stadiums as a site of opposition (Trimbur, 2019). This is the reason that we focus here on a narrower sense of the concept of geopolitics related to international relations and borders.

Thus, we focus on events for which the majority of people generally have no direct experience in their daily lives. Therefore, we can assume that events and their perceptions by political actors and the public are as much the result of their nature as the way they are framed and disseminated to public opinion by media. As a consequence of the limited place allocated to foreign news in media outlets, we can assume the existence of strong competition between geopolitical events considered worth mentioning in daily newspapers, which are generally dedicated to national audiences.

### 2.2 The International Geopolitical Agenda

To question the international dimension of crises in the media, we draw on a field of research that places international relations at the heart of its approach.

Galtung and Ruge (1965) wrote an article considered the cornerstone of international news flow theory (INFT), which provides general “rules” governing the volume of news publications.

Most of the related works have been able to determine the structural factors of the salience of an event depending on the foreign country where it took place (Wu, 2000; Segev, 2016; Grasland, 2020). For instance, Segev (2016) introduced conflict variables to analyze country coverage in different newspapers. The first group of variables introduced in the model are classical “national traits” gathering different measures of power (economy, military, population, area), but the second is “relatedness” gathering different variables of linkage (conflict, trade, common border, tourism, diaspora) and “event variables” composed of a measure of conflict intensity, a global peace index, the unemployment rate, GDP changes, and death disaster accounting.

Analyzing conflict and the geopolitical agenda is a temporal challenge for news flows theory. Historical depth of analysis was introduced to depart structural evolution from conjectural situations about some regions or countries that face exceptional violent events (e.g., wars, bombing, and terrorist attacks) during the period of analysis. These events contribute to the global changes in the analysis, which also depend on the temporal scale of aggregation of news (day, week, month, year, etc.). The temporal scale of analysis also affects the weight of some events, depending on their nature. A very brief, but intense, event (e.g., a terrorist attack) has a significant impact on the agenda at the day or week level, but is smoothed at the year scale. On the contrary, a long-running event, like a war, comprises several episodes and has a cumulative effect that increases the level of the country at the month or year scale, but is undermined at the day or week scale.

The other important element highlighted by the specialists in this field is that news competition is played out on an international scale. This means that the geopolitical crisis, even if it is related to a situated issue (e.g., migration) or place (e.g., the city of Calais, the island of Lesbos, France, and Western Europe), should be analyzed in a more general system that considers a topic as a part of the wide ensemble of news and places described in the media sphere. Therefore, the geopolitical agenda is necessarily global and even more so if we focus on international news. The international geopolitical agenda is a derivation of the geopolitical agenda of the media. The addition of “international” aims to reflect the contributions from the INFT on the mechanics of publication and competition between geopolitical topics of interest at world scale. In practice, we limit the collection to the list of states mentioned in the news rather than collecting all international organizations. This limits the number of tags collected in each language through the definition of a finite list of UN member countries. Many authors working on INFT have yet to establish such harmonized dictionaries that can be freely accessible on GitHub (Watanabe, 2018) or by request to the authors (Segev, 2016).

### 2.3 Three Topics of Geopolitical Interest for the Study of Crisis

Considering the choices made in the previous section, in particular, the adoption of a state-centric perspective, it is clear that some topics of international geopolitical interest cannot be properly addressed, such as the case of the economic crisis of 2008 (as companies may be multinational) or the climate change debate (which involves such organizations as the IPCC or UN, and various actors from civil society like Greta Thunberg). We need to choose topics that are clearly related to questions of state sovereignty. For this purpose, we select three topics of interest for a comparison of the coverage of international geopolitical crisis by media from Western Europe during the period 2014–2019.

1. **International borders.** International borders have particular relevance in this area, characterized by a process of territorial integration and the liberalization of mobility. Territorial integration led to two major crises during the period, with the crisis in Crimea and the Ukrainian conflict showing evidence of the difficulties of the EU in implementing a common foreign policy. The challenges regarding defense issues and the building of a possible common geopolitics for the EU are limited by the presence of other cooperation frameworks (e.g., NATO and the historical role of the United States as a global actor) and the lack of consensus about this topic, especially the relationship with an external actor (Russia) between the different member states. Then, Brexit has raised several border dynamics between the United Kingdom (United Kingdom) and the EU, and involving Ireland.

Borders polarize the attention of societies (Guichonnet and Raffestin, 1974). To understand this, it is necessary to consider that borders are not just lines in geographic space but are political instruments. This is what Raffestin (1980) calls the “signal border”: the borders drawn on a territory (which for many are legacies and have not changed for decades) are the geographical features for new semantics written by political actors who engage the border in their political project. The stake of the border, therefore, is no longer its outline, its erection, but its sensitization, its meaning in a system of representations, or an ideology. Therefore, the media play a fundamental role in this understanding: it is one of the public spheres in which the border signal is expressed. The border is a signal with a normative scope: It refers to a set of heterogeneous discourses that animate the public debate and define the geopolitical problems and the answers provided to them.

Amilhat-Szary (2015) proposed qualifying the new era of borders, post-Westphalian, as “mobile borders.” Thinking about mobile borders is not only of interest in the dynamics of their spatial anchoring but also in their dissemination in space and in society, and the permanent adaptation of their functions to renewed objectives and challenges. As Andreas indicated as early as 2003, “traditional border issues such as trade and migration are now inescapably evaluated through a security lens” (Andreas, 2003). This political security prism, observed in particular in the countries of the North, results in heavy border security devices to curb the so-called “unwanted” people. Borders now mainly concern traffickers, migrants, and refugees (Dubet, 2018).

This definition of the border as a security device is part of a context of a shift in migratory policies that are polarizing, increasingly restrictive with regard to a part of the world, and increasingly favorable to a migratory elite; and the identification after the attacks of the 2000s and 2010s of terrorism (World Trade Center, Bataclan,…) with its external roots. Today, contemporary borders are spaces of “preventive counter-violence.”citepbalibar_*r*_ *ace* 1988.

These brief considerations on borders lead us to ask three questions.

• How is the border topic disseminated in the media sphere?
• Where are the borders of attention?
• Is there a border crisis in Europe?

2. **International migration and the “migrant crisis” of 2015.** Migration issues are also related to European integration through the question of the borders of the EU, the management of the Schengen area, and questions related to the treatment of immigrants and the related subject of alterity (Van Houtum and Van Naerssen, 2002; Van Houtum and Boedeltje, 2009; Bernardie-Tahir and Schmoll, 2014). These questions challenge the EU policy and the representations of the political stakeholders of each member state. However, they are also the subject of public opinion at different levels and in different national contexts (Triandafyllidou, 1999; Benson, 2013).

During the last decade, the notion of “migrant crisis” has become a central issue in Western Europe. As we have seen before (*Hypothesis* section), it is highly related to the question of borders. Indeed for the countries of the EU, it has a political dimension through the management of the external borders of the EU and the construction of the Schengen area (Van Houtum and Boedeltje, 2009). More broadly, it raises the question of defining otherness through the identification of populations considered as outsiders (othering) and the spatial management of the outside (bordering) (Van Houtum and Van Naerssen, 2002).

In this case, the notion of “crisis” characterizes different types of changes.

• First, it can describe the transformation of migration and of the migration system in the southern European area. This idea has been strongly criticized by academics in the field of migration studies. The degradation of humanitarian conditions and the increasing violence on the southern European border are more a consequence of a deepening of a long-term process: The political management of the external borders of the EU as a consequence of the construction of the Schengen area since the signing of the Schengen Convention (1990) and its official implementation (1995). Thus, according to a specialist in migration studies, because this delimitation process plays out over the long term, there is no “migrant crisis.” What happened in 2015 is simply a consequence of the destabilization of the system, like the fall of the Qaddafi regime in Libya, which temporarily compromised the process of externalization of the external borders of the EU, or the phase of intensification and generalization of the conflicts in Syria and Iraq since 2013 (civil wars and the rise of ISIS).
• Nevertheless, the perception of migration in the public sphere has changed during the last decades. During this period, the year 2015 can be seen as a turning point. It was a succession of significant events that brought the issue of migration to the forefront of the public sphere: massive shipwrecks in the Sicilian Channel in the Northern hemisphere spring, the daily arrival of thousands of people on the beaches of the Greek islands in the summer, the Balkan exodus, unilateral closure of the Hungarian border in September, the need for political action, and formal political declarations of heads of states, like the “*wir shaffen das*” (“we can handle this”) of Angela Merkel (August 2015). In Western European countries [and abroad; (Leconte et al., 2019)], for the general public, it was a moment of awareness about this issue; migration issues involving European borders were no longer limited to the field of experts and became a central and recursive theme in the media. Therefore, even if there was doubt about the existence of a “real” crisis, the media portrayed it as a crisis. This is evident in the quantitative (sensitive and durable changes in the salience of migration issues during the period) and qualitative [discursive shift; (Krzyżanowski, 2018)] changes during this period.

How does the analysis of the changing coverage of “migration” in the press reveal sensible changes in the representation of this issue? In recent years, strong attention has been given to media coverage of the so-called “2015 migrant crisis” in the literature, with several reports and special issues (Bleich et al., 2015; Chouliaraki and Zaborowski, 2017; Krzyżanowski et al., 2018). These numerous works have two limitations related to the analysis of the 2015 crisis.

• Few studies offer cross-national analysis; in most cases, they are focused on a national approach (Krzyżanowski, 2018; Greussing and Boomgaarden, 2017; Caviedes, 2015) or sometimes a national comparison (Chouliaraki and Zaborowski, 2017).
• In these works, it is difficult to consider the dimension of the “turning point” of the year 2015 because most of them study a limited period of time (often a few months).

Thus, this study presents an opportunity to re-examine these two points, as follows.

• By taking a period of several years (5), is 2015 really a crucial year for the changing perceptions of migration issues?
• Are these changes similar in the five countries, or are they phase shifts between different national agendas? The corpus is interested in covering media located in countries with different situations regarding borders (countries located near the external border of EU like Italy and, to a lesser extent, France vs. countries with more distant positions, like Germany and the United Kingdom).

3. **Pandemics** are considered as a third topic. Even if it has not benefited from the same research effort by the authors as the two previous ones and was not initially planned as a research topic in the H2020 ODYCCEUS project, it offers an interesting research perspective on the concept of crisis and, as we will demonstrate, present a theoretical example of perturbation of the international geopolitical agenda at the global scale during the period of analysis (from the Ebola crisis in 2014 to the COVID crisis). Many previous research in media studies has revealed strong differences between media from “North” and “South” concerning the coverage of the Ebola crisis of 2014 (Kilgo et al., 2018; Pieri, 2019) or the Zika crisis of 2016 (Bragazzi et al., 2017; Ribeiro et al., 2018). However, the COVID-19 crisis appears to be a much more exceptional crisis that has completely modified the media agenda in 2020. Therefore, we will enlarge our period from 6 months in the *Discussion* section to analyze this exceptional case.

### 2.4 Overarching Question and Hypothesis

Our overarching hypothesis is that the media of the major Western European countries do not share the same international geopolitical agenda, despite their geographical and cultural proximity and belonging to a common political union. In a space characterized by a high level of fragmentation [diversity of languages, political fragmentation with a high concentration of states, between empires, and different histories regarding migration (Ahnström, 1993; Davies, 1998; Foucher, 1998)], there is a low probability of finding a common point of view on the geopolitical questions of borders conflicts, international migration, and pandemics. We carry out a cross-national analysis of a corpus developed according to the national distribution of media outlets between five Western European countries (France, Germany, Italy, Spain, and the United Kingdom). Do we observe common patterns beyond the different media outlets and national contexts in which they are located? Do crises like the Ukrainian crisis or the migrant crisis impact Germany, France, and Italy in the same way, considering the fact that they have been more or less concerned directly by the geopolitical consequences? Do differences observed reveal different national sensitivities?

We assume that it is possible to explore the differences in media coverage of each topic in the daily press that could lead to the emergence of a crisis. Crisis can be observed through the significant variation in the salience of a topic over time (*geopolitical timeline*: when a subject is covered and how much) but also through space (*geopolitical map*: which foreign state is associated with the topic in the news). The idea of “crisis” is often criticized by the critical streams of political geography and social sciences, as an artifact produced by the public sphere, as for the so-called 2015 “migrant crisis” (Krzyżanowski et al., 2018). Classical geopolitics and international relations are also subject to criticism because of their appetite for the “crisis talks” that have led to the weakening of the scientific validity of the notion of crisis. However, we argue that crisis could be relevant and fruitful for interpreting a crisis by the media. The term crisis commonly means “a time of great danger, difficulty or doubt when problems must be solved or important decisions must be made” (Oxford Learner’s Dictionary), sometimes associated with a traumatic dimension. However, a scholarly definition of this term leads to something more general. A “crisis” could be defined as “the turning point for better or worse in an acute disease or fever” or more generally “the decisive moment,” “an unstable or crucial time or state of affairs in which a decisive change is impending,” “a situation that has reached a critical phase” (Merriam Webster dictionary). In other words, a crisis is a form of change that considers the dynamics of a process.

Our purpose is not to interpret individual events in themselves as a crisis, but to consider that the media sphere is producing a representation of a crisis by shaping the agenda of the subject (Triandafyllidou, 2018).

Studying the evolution of media coverage of a subject of interest is a classical research topic in media studies. The studies on media hypes (Vliegenthart and Boomgaarden, 2007; Geiß, 2011; Vasterman, 2018) and attention cycles (Downs, 1972) propose, for instance, a framework for analyzing the forms and structures of changes in media coverage (e.g., percent of news dedicated to a subject). Following these works, and by targeting the case of two geopolitical subjects, we propose a critical analysis of events designated commonly as “crisis” based on an empirical analysis of the media coverage of such topics. Indeed, as demonstrated by Krzyżanowski (2018) in the case of the shaping of migration news in the Polish press during 2015, substantial changes in the manner of dealing with this issue could lead to “discursive shifts.” We identify two possible ways to study shifts, considered as forms of crisis, as follows.

1) **Quantitative changes:** relative to a sensitive increase of media coverage (salience analysis through, e.g., the percentage of news related to an event or a topic).
2) **Qualitative changes:** a transformation of the discourse about the subject (lexical, semantic, discursive shift, through the temporal analysis of the lexicon, using, e.g., temporal-oriented textometrics, or methods of world embedding).

In this study, the analysis focuses on the evolution of the salience accorded to a topic with an additional variation of the qualitative shift by introducing the notion of “spatial shift,” that is, how places mentioned in the news change during the period of observation. We focus on the exploration of the three hypotheses.

• **H1: Equivalent interest for international geopolitical topics**: each of the three topics of interest benefit from the same coverage in the broadsheet daily newspapers media of the five countries. Indeed, each country has a relatively similar size (40–80 million inhabitants) with an important colonial heritage as colonizer and an important diplomatic network.
• **H2: National synchronization of the geopolitical agenda through time**: regardless of the level of correlation between the timeline of the media outlets in Western Europe, more important differences can be observed between media of different countries than between media of the same country (Hafez, 2007)
• **H3: National organization of geopolitical maps over the full period**: regardless of the level of correlation in the list of countries mentioned in relation to geopolitical events, more important differences can be observed between media of different countries than between media of the same country.

## 3 Data and Methods

This section presents the data collection and methods, and develops a discussion of the results. More details on the technical details related to the data collection, transformation, and exploration with the TELEMAC interface are available in the **Supplementary Material**.

### 3.1 Data Collection

Our analysis is based on a corpus of broadsheet mainstream daily newspapers. Obviously, this selection does not reflect the diversity of the media sphere in each of the five countries of observation. We stand on three criteria to build the corpus.

• First, following Segev (2016), we select daily national newspapers, because they are media with a specific focus on international news as sensors of the media sphere.
• Second, we select media outlets fulfilling data quality criteria (see the **Supplementary Material**).
• Third, we analyze four broadsheet newspapers by country (available in the database of Media Cloud) to ensure a minimal diversity of editorial lines (e.g., mixing left-with right-oriented newspapers. However, the sample is further reduced to 18, because some newspapers do not fulfill the expected conditions of the minimum number of news articles.

Thus, this corpus is anything but objective or representative. It is a selection of sensors for the first exploratory work, preliminary to further investigations (Table 1).

**TABLE 1:** A sample of 18 newspapers from five countries of Western Europe.

	Media	Name	Web
1	DEU_tagspi	Tagespiegel	https://www.tagesspiegel.de/
2	DEU_frankf	FAZ	http://www.faz.net/
3	DEU_suddeu	Süd. Zeitung	http://www.sueddeutsche.de/
4	DEU_diewel	Die Welt	https://www.welt.de/
5	ESP_abc	ABC	http://www.abc.es/
6	ESP_percat	Periodico de Cat	http://www.elperiodico.com/es/
7	ESP_mundo	El Mundo	https://www.elmundo.es/
8	FRA_figaro	Le Figaro	http://www.lefigaro.fr/
9	FRA_lacroi	La Croix	http://www.la-croix.com/
10	FRA_libera	Libération	http://liberation.fr/
11	FRA_lmonde	Le Monde	http://www.lemonde.fr/
12	GBR_guardi	The Guardian	http://www.theguardian.com/uk
13	GBR_indept	The Independent	http://www.independent.co.uk/
14	GBR_dailyt	The Telegraph	https://www.telegraph.co.uk/
15	ITA_mattin	Il Mattino	http://www.ilmattino.it
16	ITA_messag	Il Messaggero	http://www.ilmessaggero.it/
17	ITA_repubb	La Repubblica	http://www.repubblica.it/
18	ITA_stampa	La Stampa	http://www.lastampa.it/

The editorial diversity in our sample is combined with a diversity of locations. Because we aim to propose perspectives from different Western European countries, we select four newspapers for each of the five countries: France, Germany, Italy, Spain, and the United Kingdom. We chose these countries because of their importance in the political life of Western Europe in the last century and the availability of data and dictionaries in the five official languages of these countries (French, German, Italian, Spanish, and English, respectively) to manage the detection of the topics.

In this study, we use a 6-year period from January 1, 2014 to December 31, 2019. In the final analysis (Discussion), we have extended this central period of interest from 6 months before and after (July 2013 to June 2020) in order to analyze contextual effects and check the stability of results. This is a relatively long period of time, which provides the depth to judge whether a change is possibly a crisis. We show that a smaller period of observation does not allow the context of an event that is cyclical or localized in time. This choice is also related to data availability and because it is a period when several major events identified as major crisis occurred in Europe (annexation of Crimea, “migrant crisis,” Brexit, etc.). A data-driven approach makes it necessary to carefully select the appropriate time period depending on the topic of analysis and the hypothesis.

We do not work on a newspaper archive but on native digital data directly produced by the media: RSS feeds. RSS feed data continuously produce elements that can be collected in real time from the feed. We use the Media Cloud database, which is a collection of news RSS feeds. It is possible to obtain not only the title of news (available freely on the Media Cloud website) but also the description part of the RSS, which is generally the first sentence of the full text of the news (available only through specific extraction).

This data source is of interest for two reasons: the quality of the collection allowed by technical means, and the standardized collection of news published on the web by each media outlet. As raw material, an RSS item includes everything needed for a spatiotemporal analysis: 1) textual content: the title of the original article published on the website of the newspaper and a description (between one and four sentences); and 2) several metadata describing the context of the publication (date and hour of publication, and the media outlet).

In the framework of this study, we proceed to a selection of news that can be considered as “foreign.” As explained in the conceptual part, *foreign news is defined from a state-centric perspective* as a piece of text (title, sentence, paragraph, and full article) related to an event happening in another country in which the media outlet is located. According to the definition proposed by Wu, foreign news provides a link between a *host country* that provides readers with information about a *guest country* (Wu, 2000). The definition of foreign news is not limited to the collection of news declared as “international” by the media outlet but can be extended to the collection of any news in which foreign countries are mentioned, regardless of the classification proposed by the media for the organization of its dissemination (“sport,” “Culture,” “Economy,” etc.,).

### 3.2 Transformation of News in Hypercube

“I keep six honest serving-men (They taught me all I knew). Their names are What and Why and When *And How and Where and Who.*”

Rudyard Kipling, The Elephant’s Child.

**FIGURE 1:**
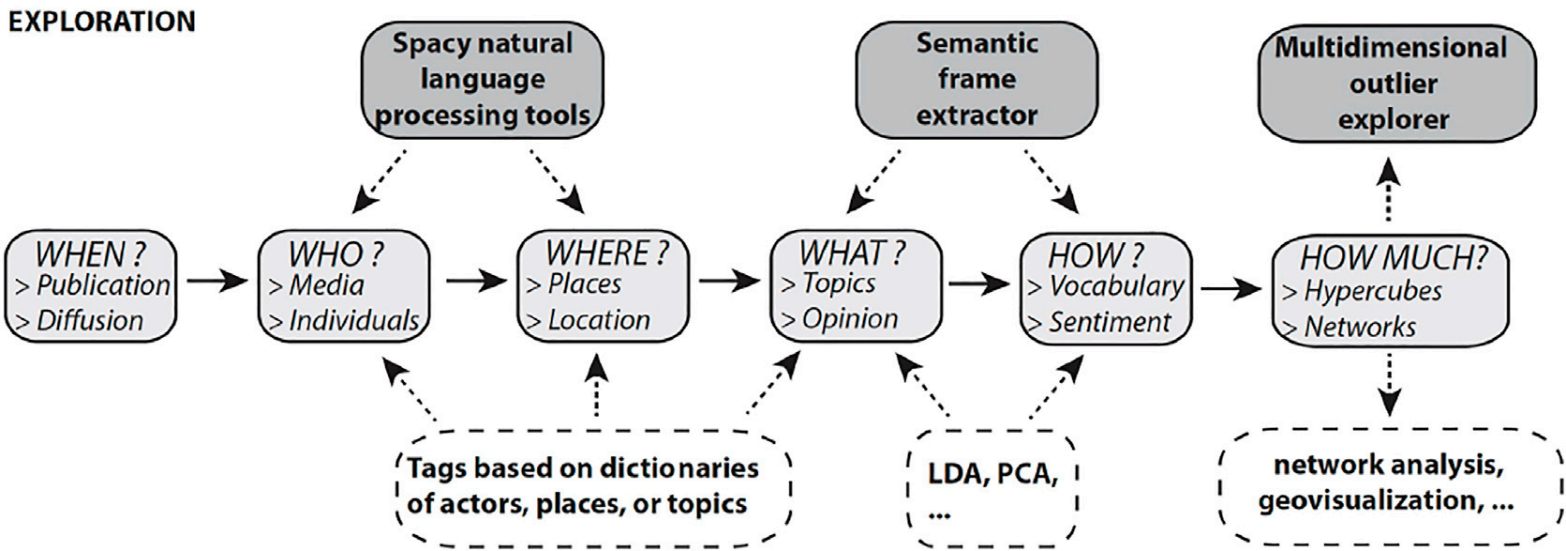
A conceptual framework for the analysis of news feeds (source: H2020 ODYCCEUS Project, Del. 2.3, Social and Spatial Interaction Models.) https://ec.europa.eu/research/participants/documents/downloadPublic?documentIds=080166e5c0561980&appId=PPGMS

As explained by Kipling, six questions are typically used to understand a problem. Of them, five (who? what? where? when? why?) are used in the school of journalism to define what should be written necessarily and as early as possible in news, in order to provide a full and complete description of an event (Lasswell, 1948). According to Wenxiu (2015), this “*5W framework*” is still relevant in the digital age. Using the work of the H2020 ODYCCEUS project, we develop this conceptual framework to elaborate a specific application for the analysis of international news, in general, and geopolitical crisis, in particular (Figure 1).

The details are developed in the **Supplementary Material**. Here, we briefly present the data model used for the elaboration of hypercubes with five dimensions than can be further combined for the exploration of data and the hypothesis testing. Each topic of interest for an observer or researcher is the starting point of a data pipeline that starts from textual units and is achieved by the creation of a data table with five dimensions, called hypercubes, which have the same time compact in terms of size, are efficient for visualization, and are free of initial copyright, as the textual data has been aggregated and transformed into counts that cannot be reversed.

• **What:** The identification of topics of geopolitical interest is based on the creation of dictionaries of keywords or regular expressions associated with the topics of interest in the five languages of our sample of newspapers. The most important issue is the choice of keywords that are associated with equivalent concepts or objects in the different languages. For example, it is relatively easy to identify topics related to specific names, such as the case of pandemics (e.g., “Ebola,” “COVID-19,” and “H1N1”). By comparison, it is more difficult to define a topic that covers a complex social reality or a fuzzy notion, as in the case of borders and migration. 1) The “border” topic is identified by the existence in each language of a specific word associated with the limit of sovereignty between two states (see the **Supplementary Material**). 2) The “international migration” topic is identified by the existence in each language of a short list of keywords that are associated with categories of migrants and refugees (see the **Supplementary Material**).
• **When:** The idea of *news* is necessarily related to the question of time, and each news item is associated with a precise day or even hour. However, daily newspapers are characterized by a weekly news production cycle: during the weekend (i.e., Saturday or Sunday), these media outlets publish 10%–30% fewer items than during the week. Empirical studies also reveal qualitative changes between the day of the week and weekends regarding the topics of interest. Two solutions exist to reduce this disruption of the weekly cycles: 1) work on data aggregated by week; 2) as in the present study, use of a more refined solution with a rolling mean of 7 days (combined with the exclusion of news published on Saturday or Sunday, according to Vasterman (2018)). Even if weeks are the most important factor of variation, other cycles play a role in the quantitative production of news: for instance, in Western Europe, the end of the year (the Christmas and New Year period) is characterized by a decrease in the number of publications and a specific kind of production (retrospectives, e.g., articles about the “news of the year”). In the Northern hemisphere summer, the holiday period in each country (or *Länders* in Germany) is characterized by a small decrease in the total number of publications, but an increase in the coverage rate of foreign news. This difference suggests that time should initially be employed on a daily basis, but with the possibility for an interface for aggregation at the upper level of weeks, months, quarters, and years.
• **Who:** The RSS feeds sent by a newspaper on the web are generally organized in different “channels” corresponding to different topics. This means that the source of news is not necessarily equivalent to a newspaper’s entire online production, but can be limited to a specialized field. In many cases, researchers have focused on selected channels specifically dedicated to foreign news (Segev, 2016; Leconte et al., 2019; Grasland, 2020). However, in the present study, we opt to use the global production of each newspaper on all channels, thanks to the Media Cloud database, which provides a global collection of all RSS channels of each media of interest. The qualification of international news, therefore, is realized *a posteriori* through the identification of textual units where foreign countries are mentioned. The identification of the country of location of the media outlets (guest country) is important, because it opens up the possibility of aggregation of isolated media toward a sample of national press. The allocation of a country to the newspapers is generally obvious because we select newspapers characterized by their national audience, that is, because the readers of the print version are distributed throughout the country where the media is located. However, two issues should be highlighted. On the one hand, we have the case of countries where the press is more decentralized than in France or the United Kingdom, and where the qualification of “national” cannot be separated from a more “regional” (i.e., infranational) dimension. This is the case for newspapers, such as *Süddeutsche Zeitung* in Germany and *Periodico de Catalonia* in Spain. On the other hand, we have the case of broadsheet newspapers with international and global audiences, especially through their websites, which provide national coverage but have a wider dissemination strategy (e.g., *The Guardian* located in the United Kingdom and *Le Monde* in France).
• **Where:** Contrary to the previous dimensions, which can be considered as metadata of the news, this dimension is the result of textual analysis of the textual content of the RSS feed (title or description). This descriptive variable is the list of the tagged countries according to the dictionary in the five languages (French, English, German, Italian, and Spanish), which detects: 1) the name of the country, 2) the name of the country’s inhabitants, 3) the adjective associated with the country, and 4) the capital city of the country (see **Supplementary material**). This dimension is crucial because it is at the same time a criterion of selection of the corpus of foreign news (we eliminate the sentences that do not mention foreign countries) but also a dimension of major geopolitical interest because it can reveal which countries are the most associated in the press with the research topic.
• **Weight:** The final dimension refers to the number of news that can be observed in each cell defined by the four previous dimensions and is used to answer to define the “Weight” I.e., the quantity of news that are observed when crossing the previous dimensions.

The number of news in the hypercube dataset is as follows: each news item (title or sentence from the description) receives a set of four attributes: 1) a unique news outlet (e.g., *The Guardian*; 2) a unique day of publication (e.g., April 1, 2014); 3) a non-empty list of foreign countries mentioned (e.g., Russia and Ukraine); 4) a Boolean test of the existence of the topic (e.g., Border = TRUE). When a single foreign country is mentioned, the news receives a weight of 1. However, when several foreign countries are mentioned, the item is divided into a fraction of news to keep the total sum of the hypercube equal to the sum of news (see **Supplementary Material** for details).

### 3.3 Exploration and Statistical Modeling

The exploration of the data is undertaken with TELEMAC, an interactive dashboard elaborated by the authors as part of the H2020 ODYCCEUS project. The technical aspects of the tool and the numerous possibilities of analysis it proposes, especially concerning the exploration spatial dimension of the geopolitical agenda, are not presented in this paper; however, some elements are available in the **Supplementary Material** https://claudegrasland.github.io/telemac/and GitHub https://github.com/ClaudeGrasland/telemac.

**FIGURE 2:**
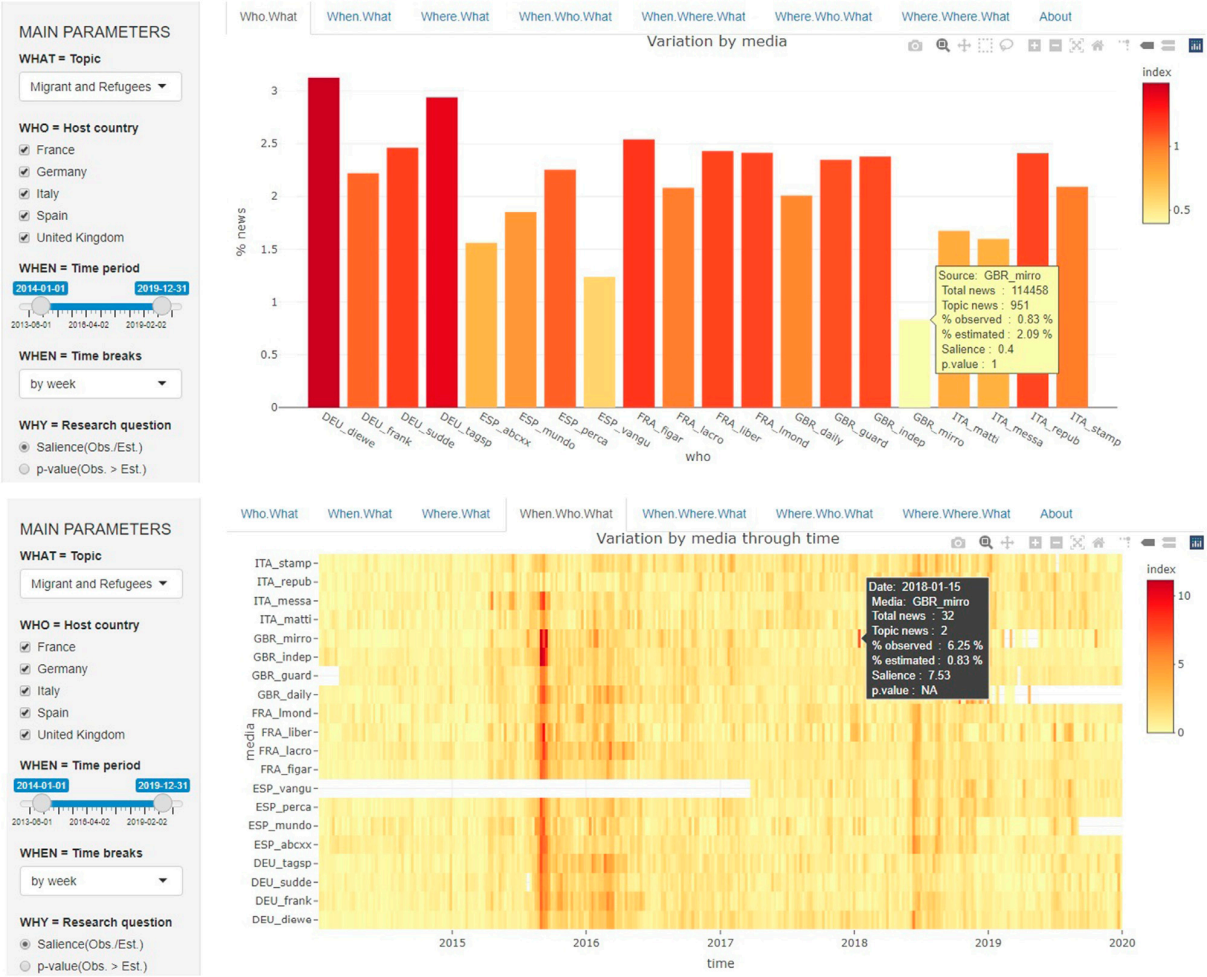
Example of the use of the TELEMAC interface for the analysis of outliers in timelines by media.

**Validation of the corpus and checking the tagging procedure** of topics and guest countries is the first interest of this interface, which makes it possible to project a complex object like the hypercube over different facets, which is easier to analyze than the whole object. Figure 2 (top) presents an example of a representation of the salience of the topic of migrants and refugees through time and by media outlet. Panel **Who. What** presents the level of salience over the period 2014–2019 and shows which media outlets have the highest salience (*Die Welt* has the highest proportion at 3.13% of foreign news) and the lowest salience (*Daily Mirror* has the lowest proportion at 0.83%) compared with the reference value of the full sample (2.09%). The comparison with other newspapers from the same country suggests that *Die Welt* is not an exception in Germany: all German newspapers are characterized by a high level of salience. However, this is not the case in every country: the *Daily Mirror* is characterized by a lower level of salience than other newspapers in the United Kingdom. This could be related to the nature of the outlet, which is often considered a tabloid when the other outlets are broadsheet newspapers. This result suggests that we should remove it from the sample. We also observe a low level of salience in the case of the Spanish newspaper *La Vanguardia*, even though it is not a tabloid.

**Choice of the relevant scale of time and media aggregation** is the second and probably the major function of TELEMAC. In the next section, we aim to test H2 by computing the correlation coefficient between the levels of salience of media over time. Salience is defined as the ratio between the observed and expected number of news items, using the average probability of each media outlet over the full period and is shown in panel **When. Who. What** in Figure 2 (middle). At the week level, we notice that data are missing for some newspapers, such as the Spanish newspaper *La Vanguardia* until April 2017, and this lack of coverage during the major crisis of 2015–2016 explains why the average level of coverage was low in the previous analysis. Therefore, this newspaper should be removed from the correlation analysis.

In conclusion, according to our explorations with TELEMAC, we exclude from the sample two newspapers with too many missing values (*La Vanguardia*) or those that do not correspond to broadsheet newspapers, which are likely to produce a geopolitical vision of the world (*The Daily Mirror*).

However, TELEMAC is mainly dedicated to exploration, and specific statistical models have been used to test our overarching question and our hypotheses.

To test H1, we use a simple model of variance analysis, whereby we compare the average level of salience of a topic for media located in the same country and media located in different countries.

To test H2, we build a correlation matrix between the levels of salience of the 20 media outlets at the month level (using pairwise complete observations when missing data are present). We compare the distribution of the level of correlation for the media located in the same country and thus, located in different countries with a variance analysis and a Fisher test. To evaluate the effect of outliers, we compare the results obtained with Bravais–Pearson and Spearman coefficients.

To test H3, we compute for each media and each topic the spatial distribution of news over the whole period, and we compute the Spearman and Bravais–Pearson coefficient of correlation between these different distributions. We exclude the five host countries (where the media is located) from all spatial distributions to obtain a better comparison. Then, we use the same methodology as for the timelines and proceed to a variance analysis to compare the degree of similarity of geopolitical maps between media located in the same country versus different countries.

## 4 Results

This section tests our hypotheses about the possible preeminence of the national effect on the geopolitical agenda. Can we observe the same level of salience in the coverage of the topics for newspapers located in the same country (H1)? Can we observe the same pattern in time (H2)? Can we observe the same pattern in space (H3)?

### 4.1 Equal Salience of Topics by Country (H.1)

**FIGURE 3:**
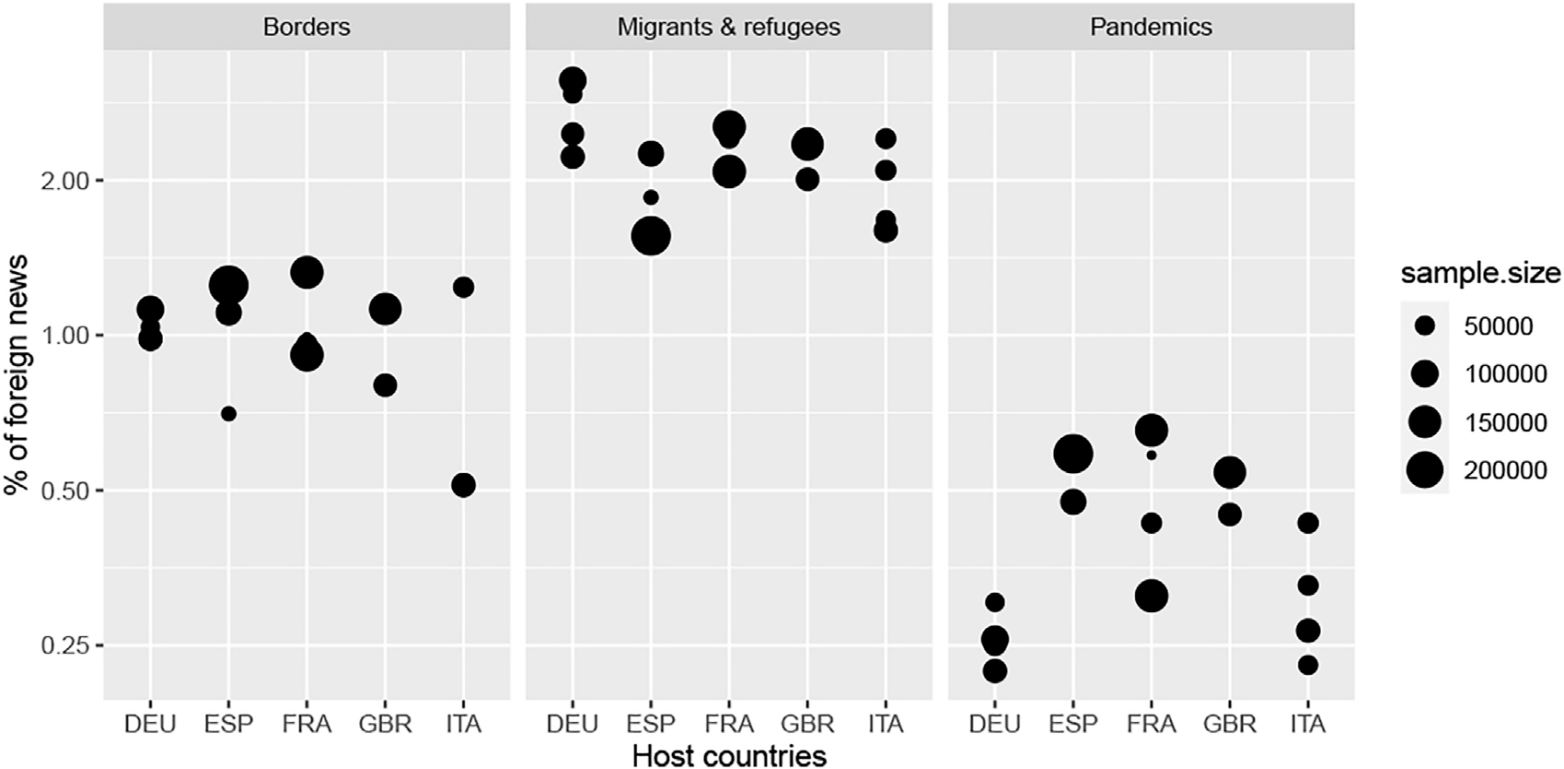
Salience of topics by host country.

The analysis of the salience of each topic by media does not reveal always significant differences between host countries for the period of observation **(** Figure 3 and Table 2).

• **The topic of borders** is generally present in approximately 1% of the foreign news (*mean* = 0.96*%*, *median* = 0.99*%*) with small variations between media outlets (*CV* = 0.27), except for the case of Italian newspapers, where the proportion is generally small except in the case of *La Stampa.* We conclude that nationality of media outlets does not influence this topic (*p* = 0.246).
• **The topic migrants and refugees** has the highest level of salience of the three topics: 2% of foreign news (*mean* = 2.2, *median* = 2.3). It also has lower variability than the borders in relative terms (*CV* = 0.19). The salience of the topic is generally higher in Germany and lower in Spain, and produces significant differences between all nations (*p* = 0.032).
• **The topic of pandemics** is the least mentioned during the period of observation 2014–2019 (*mean* = 0.41, *median* = 0.43) with important variations between media in relative terms (*CV* = 0.35). This weakness is probably related to the long period of observation chosen and the fact that period does not include the year 2020, when 20–40% of the news related to the words “COVID-19” or “coronavirus.” The differences between newspapers located in the same country and in different countries are very significant (*p* = 0.003), with a contrast between countries with lower coverage (Italy and Germany) and those with higher coverage.

**TABLE 2:** Testing the hypothesis of equal salience of topics by host country.

	*Dependent variable*		
—	Border (1)	Migrants, refugees (2)	Pandemics (3)
Spain	−0.011	−0.799***	0.296***
—	(0.183)	(0.251)	(0.071)
France	0.017	−0.321	0.237***
—	(0.170)	(0.232)	(0.066)
United Kingdom	−0.013	−0.442	0.251***
—	(0.183)	(0.251)	(0.071)
Italy	−0.338*	−0.744***	0.055
—	(0.170)	(0.232)	(0.066)
Constant (Germany)	1.032***	2.687***	0.259***
—	(0.120)	(0.164)	(0.047)
Observations	18	18	18
R^2^	0.323	0.531	0.690
Adjusted R^2^	0.114	0.387	0.594
Residual Std. Error (df = 13)	0.240	0.328	0.093
F Statistic (df = 4; 13)	1.549	3.685**	7.220***

Note. *p < 0.1; **p < 0.05; ***p < 0.01.

As a whole, we cannot validate or reject H1, as the results clearly depend on the topic of interest. However, it should be noted that each topic is characterized by a specific level of interest over the period with relatively limited variations in the sample of the 18 newspapers observed. In each media, the most important geopolitical topic is the question of migrants and refugees, followed by borders, and finally, by pandemics.

### 4.2 National Synchronization of Geopolitical Agenda Through Time (H2)

The second hypothesis is about the temporal dimension of the geopolitical agenda. We assume that, all things being equal in the global level of interest for a geopolitical topic, the distribution of the period of interest is influenced by the national agenda, which produces significant differences in the timeline of the topic over the period of observation.

**FIGURE 4:**
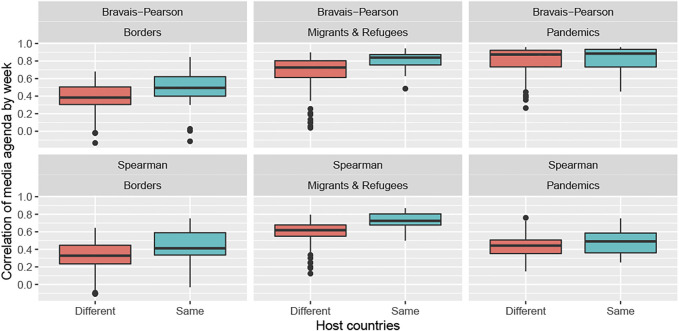
Impact of the nationality of the media on the synchronization of their geopolitical agenda (2014–2019).

The results support the hypothesis of synchronization for the topics of borders and the topic of migrants and refugees, but not necessarily the topic of pandemics (Table 3). In this last case, the national effect is confirmed only by the Spearman coefficient. This result means that the synchronization of all media at the national level is not completely perfect. Figure 4 confirms the existence of many exceptions and important differences between the topics.

• **The topic of borders** is more synchronized between the media of the same country (*r* = + 0.48, *ρ* = + 0.42) than between media of different countries (*r* = + 0.38, *ρ* = + 0.33). However, the level of correlation remains low. Major events, such as the annexation of Crimea and Brexit, have apparently not produced common hype that is likely to increase the level of correlation at the European scale.
• **The topic of migrants and refugees** appears to be much more synchronized at the European level than the border topic, even if the synchronization is higher between media of the same country (*r* = + 0.81, *ρ* = + 0.73) than between media of different countries (*r* = + 0.67, *ρ* = + 0.59). This high level of correlation is clearly induced by the significant increase in the salience of the topic in all Western European countries in 2015 and by a lower level of coordination, such as the crisis in 2018 involving the Aquarius rescue vessel.
• **The topic of pandemics** presents a more complicated pattern with stronger heterogeneity in terms of time distribution, with a major spike in 2014 (Ebola outbreak in Western Africa), a secondary spike at the beginning of 2016 (Zika outbreak in Brazil) and several smaller spikes during most of the period. This explains the divergence between the results obtained with the Bravais–Pearson coefficient (high level of correlation but few differences between countries because all of them are affected by the two major crises) and the Spearman coefficient (lower level of correlation but more synchronization at the national level owing to the same coverage of minor events).

**TABLE 3:** Testing the hypothesis of national synchronization of the geopolitical agenda through time.

	*(a) Bravais–Pearson correlation*		
—	Borders	Migrants and refugees	Pandemics
Same country	0.101***	0.137***	0.006
—	(0.029)	(0.028)	(0.027)
Constant (different country)	0.380***	0.670***	0.796***
—	(0.012)	(0.011)	(0.011)
Observations	306	306	306
R^2^	0.037	0.074	0.0001
Adjusted R^2^	0.034	0.071	−0.003
Residual std. error (df = 304)	0.186	0.176	0.171
F Statistic (df = 1; 304)	11.811***	24.445***	0.042
—	*(b) Spearman correlation*		
—	Borders	Migrants and Refugees	Pandemics
Same country	0.090***	0.132***	0.048***
—	(0.027)	(0.020)	(0.018)
Constant (different country)	0.327***	0.595***	0.436***
—	(0.011)	(0.008)	(0.007)
Observations	306	306	306
R^2^	0.036	0.127	0.023
Adjusted R^2^	0.032	0.124	0.019
Residual std. error (df = 304)	0.172	0.126	0.115
F Statistic (df = 1; 304)	11.210***	44.186***	7.033***

Note. *p < 0.1; **p < 0.05; ***p < 0.01.

In conclusion, the topic of migrants and refugees stands out from the other two topics because of the high level of temporal synchronization between the media located in different countries. On the contrary, national agendas seem to be more visible in the case of the topics of borders and migration than for pandemics, even if the topic of pandemics has a more complex temporal pattern structure.

### 4.3 National Organizations of Geopolitical Maps Over the Period 2014–2019 (H3)

The spatial dimension of the geopolitical agenda is related to the foreign countries mentioned in news related to a topic. The relatively long period of observation leads to a more structural view than the results for shorter periods, which are mechanically more event oriented. For instance, all the media outlets mentioned Turkey and Greece by talking about migrants and refugees in September 2015: the climax of the so-called “migrant crisis.” However, it is not obvious that all these media outlets mentioned these countries in relation to this topic before or after this period of maximal interest. Furthermore, it is not obvious that the media of all countries provided equivalent media coverage to other events related to this topic, like the “Rohingya crisis” (since 2016) and the Trump administration’s decision to build a wall between Mexico and the United States (2017–2018). According to, among others, Ostgaard (1965), the relative distance of these events from the country where the outlets are located would probably lead to less coverage of these events. This is more so compared with Euro-Mediterranean-centered news, like the “2015 crisis.”

**FIGURE 5:**
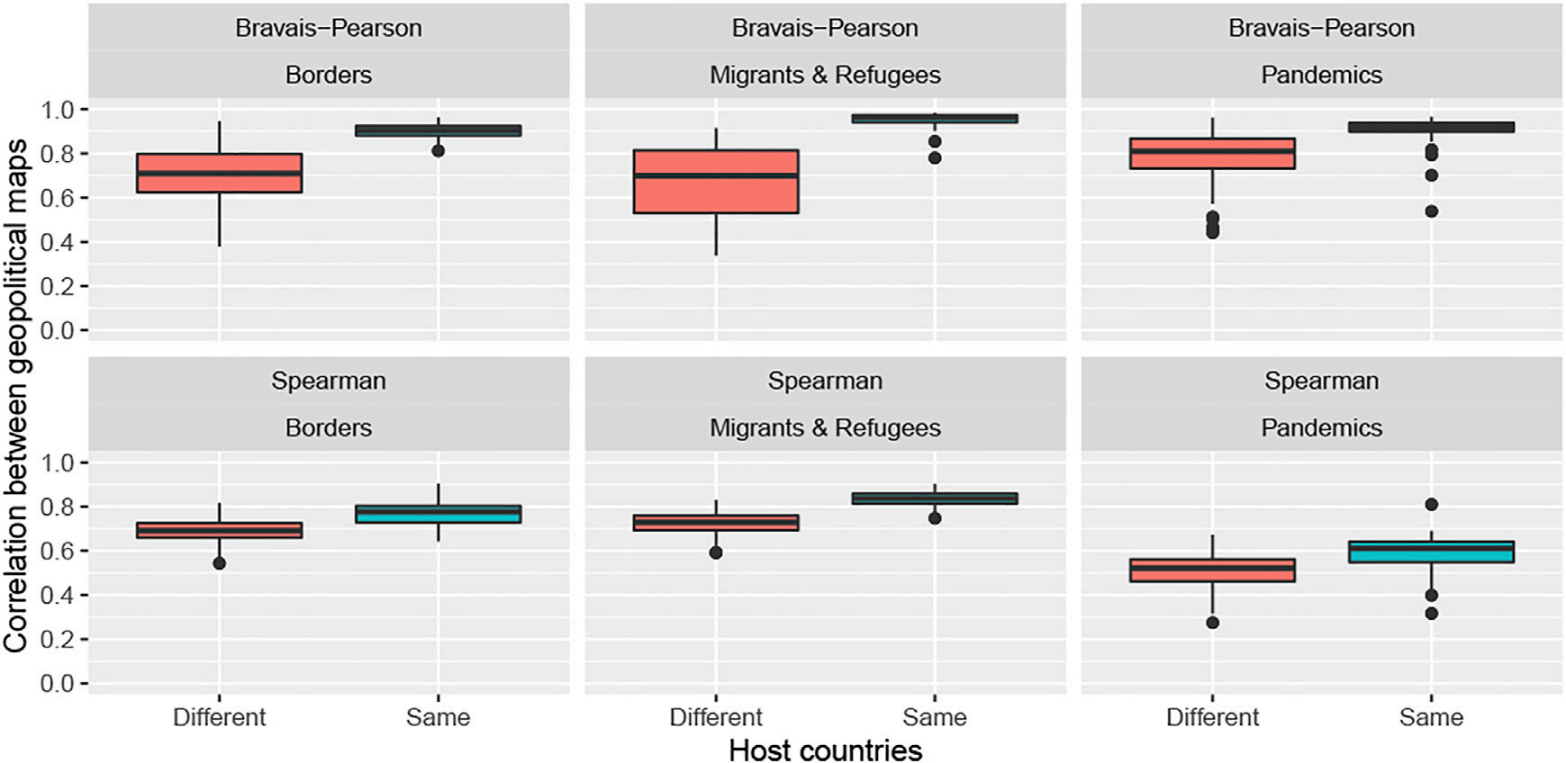
Impact of the national belonging of the media on the spatial organization of their geopolitical agenda (2014–2019).

**FIGURE 6:**
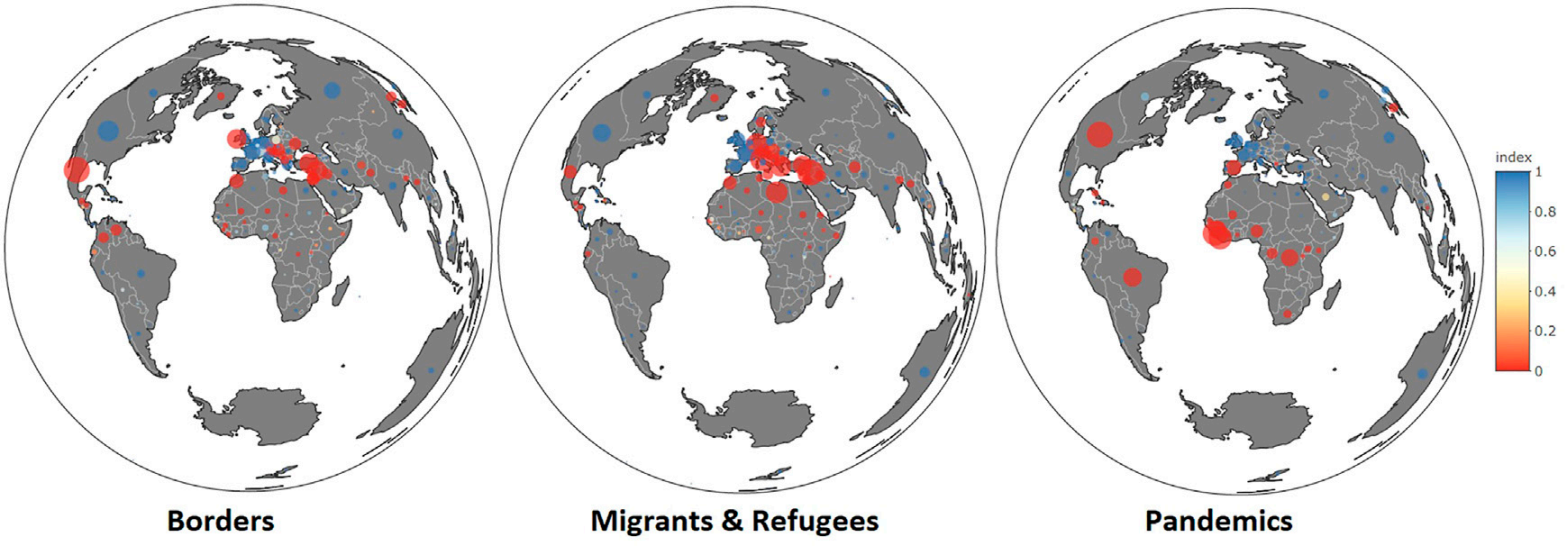
Map of media coverage of three geopolitical topics in the Western European press (2014–2019).

The results presented in Figure 5 and Table 4 confirm H3, with a very high level of significance, for each case study. The interest of media in a geopolitical event is strongly related to the location of events, according to the rules of selection of foreign news established by Galtung and Ruge (1965). Figure 6 presents a visualization of the countries that are the most significantly associated with each topic of interest.

• **The map of topic borders** is characterized by a focus on news located at the border of the EU (Ireland, Ukraine, and Turkey) or in its southern and eastern neighborhood (Morocco, Libya, Syria, etc.). However, the coverage of the border topic is also important in Latin America (Mexico and Venezuela), sub-Saharan Africa, southern Asia, and Korea. Countries commonly identified as major geopolitical actors (Russia, China, and the United States) are associated with a large amount of news about borders (size of the circle). However, they are not specifically associated with the topic because they are the subject of many other stories related to other topics (sport, trade, economy, etc.).
• **The map of the topic of migrants and refugees** shows a concentration of news in the Balkans, northern Africa, and the Middle East. It is related to the period of maximal attention commonly seen as the turning point of the “migrant crisis” between the Northern hemisphere summer of 2015 and early 2016. Other events of the period, such as the Rohingya crisis and the edification of Trump’s wall at the border between the United States and Mexico are visible but less important.
• **The map of the topic of pandemics** is strongly structured by two major pandemics: Ebola (2014) and Zika (2016). Countries associated with the topic are mainly located in the global South (countries of origin of the outbreak: Liberia, Guinea, Sierra Leone, and Brazil). Some Northern countries concerned with dissemination of this news are also represented (the US and Spain). Surprisingly, very few countries from eastern and southern Asia are mentioned during this period, even though they were the sites of many outbreaks or risks. Again, the results would likely be different if the period had included the year 2020.

**TABLE 4:** Testing the hypothesis of national structure of the geopolitical agenda in space.

	*(a) Bravais–Pearson correlation*		
—	Borders	Migrants and refugees	Pandemics
Same country	0.198***	0.280***	0.107***
—	(0.019)	(0.024)	(0.017)
Constant (different country)	0.703***	0.668***	0.784***
—	(0.007)	(0.009)	(0.007)
Observations	306	306	306
R^2^	0.271	0.310	0.111
Adjusted R^2^	0.269	0.308	0.108
Residual std. error (df = 304)	0.118	0.152	0.111
F statistic (df = 1; 304)	113.038***	136.735***	37.808***
	*(b) Spearman correlation*		
—	Borders	Migrants and Refugees	Pandemics
Same country	0.078***	0.108***	0.076***
—	(0.008)	(0.008)	(0.013)
Constant (different country)	0.690***	0.722***	0.511***
—	(0.003)	(0.003)	(0.005)
Observations	306	306	306
R^2^	0.233	0.382	0.104
Adjusted R^2^	0.230	0.380	0.101
Residual std. error (df = 304)	0.051	0.050	0.081
F statistic (df = 1; 304)	92.194***	188.060***	35.401***

Note. *p < 0.1; **p < 0.05; ***p < 0.01.

### 4.4 Fluctuation Between National and European Synchronization Over Time

**FIGURE 7:**
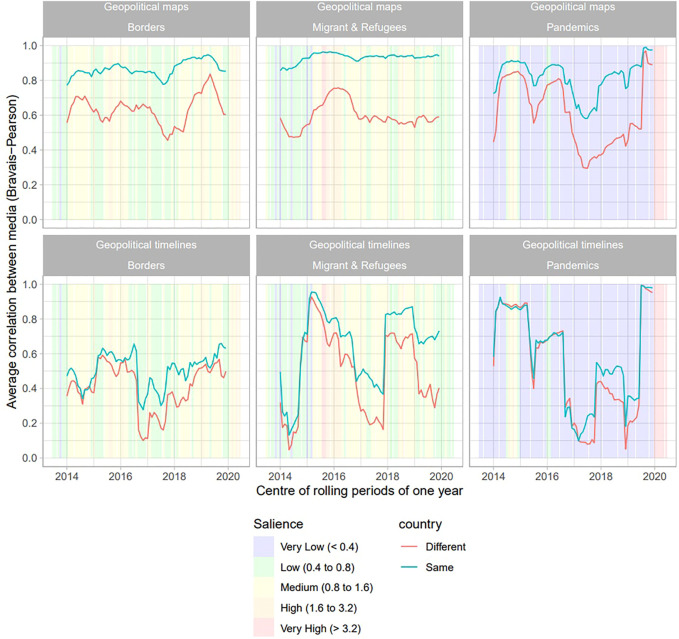
Analysis of spatial and time synchronization of the media agenda by rolling periods of 12 months.

To assess the impact of periods of crisis on our initial hypothesis, we replicate the analysis of time correlation (H2) and spatial correlation (H3) for a shorter period of 12 months for all 720 months of the period of analysis. This disaggregation of the model by rolling periods implies an extension of the time period of 6 months before and after the initial period of analysis, as the measurement conducted, for example, in January 2014, implies the collection of data for July–December 2013 and the measurement conducted in December 2019 is for January–June 2020 data. The results (Figure 7) reveal strong variations in the synchronization of media agendas in space, as in time. Specifically, the results support the hypothesis that the convergence between media increases during periods of crisis, and this convergence produces a reduction of differences between media of the same country and media of different countries. Certainly, H2 and H3 hold on average for a long period of time. However, the conclusion would probably not be the same if the analysis involved a shorter period of time. In a previous study, authors conclude there is global convergence of the media agenda at the world scale concerning the topic of migrants and refugees during the year 2015. However, their conclusions would certainly have been different if they had chosen the year 2018. This result means that it is of utmost importance to precisely analyze the effect of specific events on the geopolitical agenda of media.

## 5 Discussion

In this section, we first bring to light the global results of this study, that is, the national versus European dimension of crisis. Second, we explain how this study contributes to the understanding of how each of the three topics is framed in the media sphere. Finally, we will discuss some perspective for the causal explanation of the results: to what extent it is possible to precisely explain the convergence and divergence of the geopolitical agenda between the five countries of Western Europe.

### 5.1 Global Results: Crises are First Set at the National Level but European Moments of Synchronization Exist

In conclusion, the results of this study are as follows.

• There is a comparable level of salience for each topic in the five countries.
• There is national synchronization of the geopolitical agenda when a topic is covered by the media outlet.
• We provide clues of national organization of geopolitical news maps (the countries mentioned change in each country where the media outlet is located).
• There is strong synchronization within each national context when the salience of a topic increases. These moments can be identified as moments of possible crisis at national scale.

This study works on equivalent keywords in the five languages of each country to detect the topics of interest, identify and compare guest countries, and perform a cross-analysis. Doing so makes the results comparable and allows us to question the differences of perceptions across cultural contexts in Western Europe, which is commonly perceived as a coherent cultural and political area.

That the focus on the crisis reveals a national agenda is not a trivial result. It confirms that agendas remain at the national level in the context of Europeanization. However, the analysis of dynamics over time shows a more important result: situated agendas can be both framed and synchronous at the national and regional levels. As a mixture of these two levels, we observe in the results a pre-eminence of the national level with some regional exceptions, especially during the moment identified as a crisis.

A crisis seems to be moments of synchronization of the geopolitical agenda at European scale. It could be interpreted as evidence of an emergent European public sphere. As mentioned by Moisio et al. (2013): “A contextually grounded analysis of the Europeanization of popular culture and the media could allow us, for instance, to elaborate new understandings of an emergent European public sphere that move beyond “still-national” conceptions.” Perhaps crises play a driving role in the emergence of these over-national conceptions. In addition, this is even more possible in the member states of the UE: a political entity that leads to common concerns, especially on issues related to EU border policies, such as international migration and border conflicts in Eastern Europe or pandemics. This idea is, at this stage, only a hypothesis and probably needs to be tested. This may be due to the larger selection of topics and replication of the method on other media outlets and periods of analysis.

### 5.2 Results by Case Studies

We briefly discuss the specific results obtained for each topic according to the state of the art.

#### 5.2.1 Borders in Press (2014–2019): Weak Europeanization of the National Geopolitical Agenda

**FIGURE 8:**
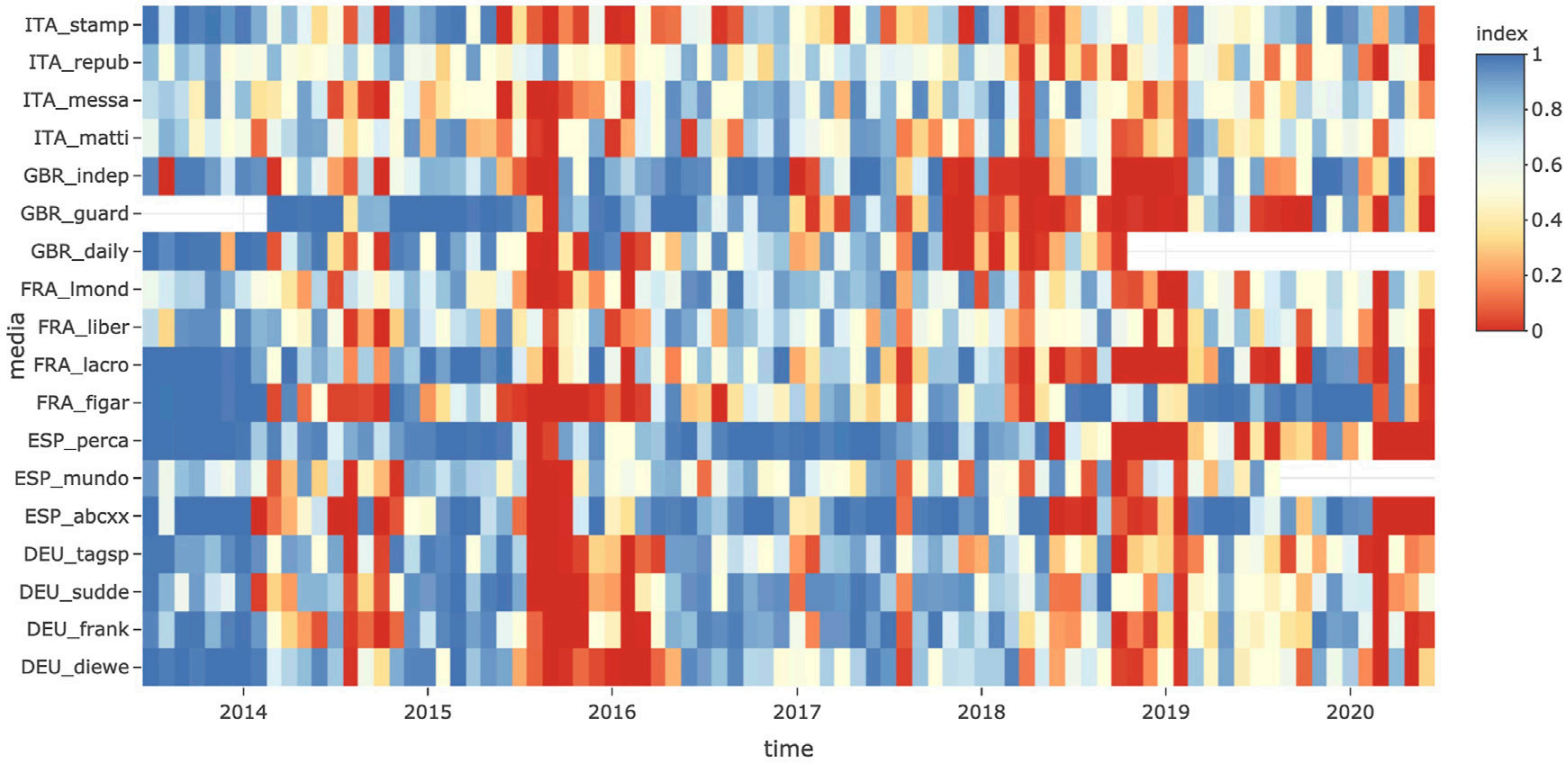
Media coverage of the border topic in the foreign news of 18 newspapers from July 2013 to June 2020.

Based on the results, we highlight the contribution of this case study and interpret the general timeline of the news revealed by TELEMAC (Figure 8).

First, unlike migration, this topic has a poor level of timeline synchronization but quite a good level of map synchronization. In other words, European newspapers of the corpus are unanimous on places to cover, but vary on the moment and intensity of coverage. This is a specificity of this topic, which is related to the nature of borders. Borders are a political topic whose spatial dimension is well defined: it refers to a line on the ground that delineates two countries that are recognized by most of the international community. There is a type of spatial ambiguity. This is not the case for events as reflected by the timeline synchronization patterns. Contrary to migration or pandemics, the border topic synchronization does not evolve a shock pattern. A sudden synchronization can be imagined as the result of a trigger. In this case, there are no evident triggering moments. This may be linked to the nature of border events that are not well bounded in time, for example, the Northern Ireland border deal within the Brexit negotiations. This has been a long process spread over time. The same holds for the US–Mexico wall of Trump or the recurrence of the Syrian conflict. The lasting nature of border events can encourage journalists to recount them at different moments.

This conclusion does not hold for 2015, which was a time of higher Europeanization of timelines (good synchronization), but is a moment of lower Europeanization of maps. This is because of events related to the border topic at this time, when the border topic merged with the migration topic. However, migration at borders involved the own borders of reporting countries and direct relations with neighbors. Thus, the degree of involvement of the countries of the corpus had an impact on the level of synchronization. At this time, French newspapers might have covered Italy extensively, because their common border was at stake, whereas Germany covered Austria extensively for the same reason.

On the contrary, the end of 2019 had high synchronization of map and timeline: it is possible that this period had two synchronization parameters. These were the sudden onset of the COVID-19 pandemic and, a distant event that did not directly involve the reporting country, namely, China.

We conclude as follows.

• A general topic, such as borders, addresses events of different natures regarding their suddenness and their remoteness, leading to different degrees of synchronization at the European scale.
• Synchronization of agendas must be analyzed by taking different dimensions as space and time, because it may be observed in one dimension and not another. Consequently, one should add a qualitative dimension to qualify the content of news and evaluate lexicon synchronization.
• The border agenda is weakly European and with a prevalence of coverage at the national level.

#### 5.2.2 The “migrant crisis” of 2015: a complete crisis?

**FIGURE 9:**
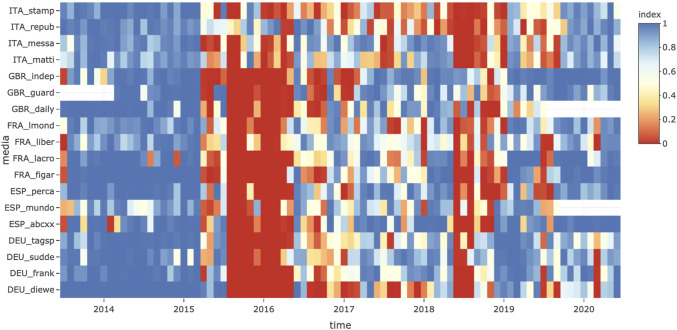
Media coverage of the migrants and refugees topic in the foreign news of 18 newspapers from July 2013 to June 2020.

First, if we look at the global coverage of the topic related to migrants and refugees in the whole corpus (Figure 9), the year 2015 is undeniably a crucial one. The record of salience of the topic for the whole period is in the Northern hemisphere summer 2015, during which time the media coverage of migration is nine times higher than the average of the period of the previous period (1%). This spike is followed by a structural change with an increase in the average coverage after May 2016 (closer to 2%). Thus, the conditions for a crisis are met: we observe that in 2015, there was a sharp increase in focus on migration, followed by a sustained increase in average attention.

The evolution of this salience confirms the importance of 2015 as a turning point. It was a time of high salience and high synchronization of timelines and geopolitical maps although it is important relative to the second period of attention in 2018. However, 2018 is not as synchronous as 2015. Timelines and geopolitical maps vary according to the country of observation.

This can be explained by the fact that the 2018 “migrant crisis” was first and foremost an Italian crisis. This spike is related to the Aquarius episode: in the European summer of 2018, several countries refused access to their harbors to the humanitarian ship of the NGO “SOS Mediterranée.” This period was a period of intense controversy concerning the responsibility of states for migrant rescue in the Mediterranean Sea and, in particular, over the central role of the Italian government in this issue, and the anti-immigrant politician Matteo Salvini used this event and migration as a central issue in his electoral campaign.

Obviously, national agendas are not synchronized: preferences emerge, and they are mainly related to the fact that countries do not have the same role and are not in the same position regarding these issues during the period.

Another interesting finding is related to the spatial visualization of news related to migration. The main countries associated with the general idea of the “migrant crisis,” such as Turkey and Syria, play a central role in the geography of migration during this period. However, other countries also play a significant role in the geographical representation of migration issues. The US, for instance, is the most quoted country related to the topic. It has played a central role in the picture, especially related to the question of the Mexican border after the election of Trump. In addition, other countries, such as Afghanistan and Libya, are significantly associated with this topic during the period, but they are more regularly associated with migration issues, even if they were not the most quoted in 2015. In these countries (one a country of origin of numerous asylum seekers, the other a state involved in the externalization process of EU borders), the crisis became normalized and became a regular topic. These elements highlight the countries hidden behind the exceptional coverage of the Mediterranean crisis.

We conclude as follows.

• The organization of the news agenda seems to be structured at a national scale.
• These national agendas are significantly different. This point could explain the difficulties in proposing a common politics (geopolitics) at the level of the EU on the issue of migration.
• The crisis did not end in 2015. There was also a second focus on the migration issue in 2018, and it may become a focus again.
• There are several “migrant crises” in the media, not just in the Mediterranean area, and they are sometimes covered in a less exceptional manner over a longer period. Focusing attention on a limited list of places and events contributes to archetypes and leads to a kind of invisibility of issues that are less represented in the media.

#### 5.2.3 Pandemics: A Topic of Global Interest at World Scale

**FIGURE 10:**
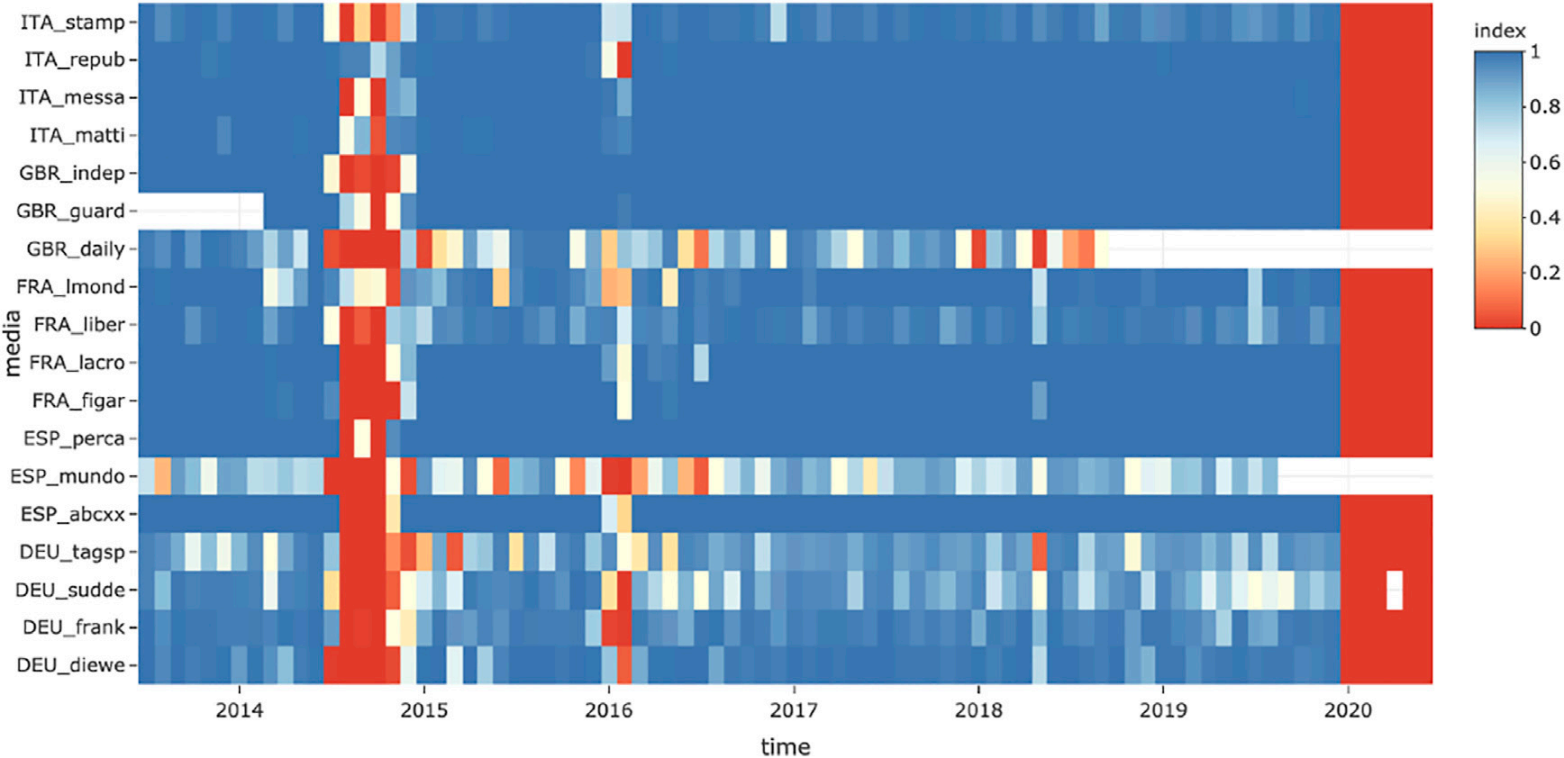
Media coverage of the pandemic topic in the foreign news of 18 newspapers from July 2013 to June 2020.

Even without considering the exceptional event of the COVID-19 crisis, it is clear that the pandemics are characterized by a very specific agenda that can be characterized as more global than European or national. The monthly coverage pattern from July 2013 to June 2020 is characterized by a very limited number of powerful instances of hype that are covered by all media at the same time (Figure 10). As mentioned in the initial part of this paper, this topic is added for comparison. After the analysis, we conclude that it is not comparable, as it belongs to a different category of world events.

### 5.3 Discussion: Three Possible Causal Hypotheses

To interpret the patterns of convergence and divergence observed for the two first topics (excluding the topic of the pandemic, which is a different category of event), we propose three causal hypotheses.

*The nature of geopolitical events:* Events that are bounded in time and space and refer to a limited set of actors are easier to grasp and can create synchronization. This refers to what Galtung and Ruge called “unambiguity”: “events which are easy to grasp make for better copy than those which are open to more than one interpretation, or where understanding of the implications depends on first understanding the complex background to the event” (Galtung and Ruge, 1965). This explanation could apply to the three case studies. First, for the “migrant and refugees” topics, because the period 2015–2016 was a moment of reduction of the word, a complex problem was localized in precise points and portrayed in sometimes very illustrative pictures (e.g., the picture of Syrian refugee child Ayland Kurdi, which went viral on Twitter and then on many front pages of daily newspapers in Europe). These moments of simplification can also be found for the border topic, such as the closure of the Hungarian border in 2015 or the media coverage of Trump’s decision to build a wall on the US–Mexican border.

*The degree of implications for geopolitical events:* Countries that are not directly involved in a geopolitical crisis are more likely to display similar patterns than those directly involved as actors or locations where events are taking place. This idea is sustained among others by the works of Segev (2016) who use variables of “relatedness” gathering different variables of linkage of countries (existence of a former conflict, trade, common border, tourism, diaspora) to explain whether news is covered more in one country than another. This hypothesis conforms somewhat with our interpretation of the events related to the Aquarius crisis for the Italian press regarding the case study of “migrant and refugees.” Regarding border conflicts, the consequence of Brexit for Northern Ireland could explain the importance of border-related issues to Brexit in the United Kingdom.

*The proximity of media to the geopolitical events reported.* Countries that are located at shorter distances, have a common language, or share a common history are more likely to be mentioned by the media of the country where newspapers are located. Divergence is particularly visible when two international geopolitical events compete during the same period (Segev, 2016; Grasland, 2020).

## Data Availability

The original contributions presented in the study are available at: https://claudegrasland.github.io/telemac/. Further inquiries can be directed to the corresponding author.
